# The Role of Serum Albumin Dynamics in the Healing of Simple Diaphyseal Tibial Fractures: A Prospective Observational Cohort Study

**DOI:** 10.7759/cureus.100047

**Published:** 2025-12-25

**Authors:** Fayaz Hussain, Faheem Ullah Khan, Akbar Shah, Syed Ahson Abbas, Anam I Satti, Shabbar H Changazi

**Affiliations:** 1 Orthopedics and Traumatology, Provincial Headquarter Teaching Hospital Gilgit, Gilgit, PAK; 2 Surgery, Provincial Headquarter Teaching Hospital Gilgit, Gilgit, PAK; 3 General Surgery, Provincial Headquarter Teaching Hospital Gilgit, Gilgit, PAK

**Keywords:** diaphyseal tibia, fracture malunion, fracture union, serum albumin, tibial diaphyseal fractures

## Abstract

Introduction

Diaphyseal tibial fractures are frequently encountered in clinical practice owing to the bone’s relatively subcutaneous position. These fractures occur along the tibial shaft between the knee and ankle joints and typically result from direct or indirect forces. This study was planned to determine the role of serum albumin in the healing of these fractures, thereby improving the overall management of patients with diaphyseal tibial fractures.

Materials and methods

This observational cohort study was conducted in the Department of Orthopedics, Provincial Headquarter Teaching Hospital Gilgit, from March 2022 to September 2025. A total of 110 patients with simple diaphyseal tibial fractures were included in the study. A detailed clinical history was obtained, a physical examination was performed, and a blood sample was subsequently drawn for the analysis of serum albumin levels. Patients were assessed for fracture union. Changes in serum albumin were compared between patients with normal union and those with impaired union of tibial diaphyseal fractures. All collected information was entered into the proforma.

Results

The mean age of the patients was 35.29 ± 11.77 years. The frequency of impaired union among patients treated for simple diaphyseal tibial fractures was 16.36% (18/110). A significant difference was observed in the mean change in serum albumin levels between the two groups. Patients in the Union Group demonstrated a positive mean change (0.24 ± 0.21 g/dL), whereas those in the Impaired Union Group exhibited a negative mean change (-0.40 ± 0.15 g/dL).

Conclusions

A changing pattern of serum albumin in the post-injury period is a strong predictor of healing outcomes in simple tibial fractures. A declining albumin level signals impaired union, whereas a rising level indicates healthy union.

## Introduction

Due to their relatively subcutaneous location, diaphyseal tibial fractures are commonly seen in routine clinical practice [1,2]. Tibial diaphyseal fractures refer to fractures that occur along the shaft of the tibia between the knee and ankle joints [3,4]. In general, the incidence of tibial shaft fractures is 16.9 per 100,000 people per year, with male dominance at 21.5 per 100,000 people per year, compared with 12.3 per 100,000 in women [5]. The tibia is particularly susceptible to complications due to its subcutaneous location and challenging blood supply. These adverse factors contribute to an increased risk of infection, nonunion, neurovascular injuries, and compartment syndrome. After the initial period, nonunion, delayed union, and malunion may add to the problem [6,7]. Fracture healing is an exceptional and complex physiological process that is affected by multifactorial components, including the poor nutritional status of patients [8].

Serum albumin is an illustrious proliferative factor in cell culture, having the propensity to stimulate mesenchymal stem cell growth on the surface of bone allograft. Albumin is a 66-kDa protein secreted from diaphyseal tissues during the fracture union process [9]. Few studies have demonstrated the association of fracture outcomes with serum albumin and have shown the anabolic effect of albumin on bone components as well as fracture healing in both in vitro and in vivo experiments. Ali et al., in their study, showed that albumin levels of less than 3.5 g/dL had a strong association with delayed or impaired fracture healing. Patients with lower levels of albumin exhibited a 4.6 times lower recovery rate, while patients with normal levels of serum albumin demonstrated early recovery, probably by enhancing the proliferation of stem cells in bone [10]. Ali et al., in another study, determined the frequency of impaired healing in patients with simple diaphyseal tibial fractures and quantified the serum albumin level and its correlation with fracture healing progression. Their results reflected that impaired healing was observed in 24% of patients. Fifty-four percent of patients presented with left tibial fractures. Road traffic accidents were the most frequent cause of injury at 64%, followed by falls from height at 24%. The mean serum albumin levels were significantly higher in the Union Group (3.58 ± 0.21) compared with the Impaired Union Group (3.43 ± 0.25), with a p-value of 0.045 [11].

Tibial diaphyseal fractures are common in our setting due to the high incidence of road traffic accidents and other relevant factors. It is of extreme importance to understand the rate of successful union of these fractures and the factors associated with impaired healing in these patients. Some previously published studies have demonstrated a strong association between serum albumin and malunion of diaphyseal tibial fractures. The present study is planned to determine the role of serum albumin in the healing process of these fractures. If significant results are obtained, monitoring of this biomarker may be advised during the healing process. This may improve the overall management of patients with diaphyseal tibial fractures.

## Materials and methods

This observational cohort study was conducted in the Department of Orthopedics, Provincial Headquarter Teaching Hospital Gilgit, from March 2022 to September 2025. Patients aged between 18 and 60 years of either sex presenting with simple diaphyseal tibial fractures were included in the study. Patients presenting with osteoporotic fractures, multiple traumatic fractures, a history of chronic smoking, uncontrolled diabetes, or steroid therapy were excluded from the study. A sample size of 110 was calculated by taking a confidence interval of 95%, an absolute precision of 8%, and an anticipated population percentage of 24% [11]. Permission and approval for the study were sought from the hospital ethics committee.

Patients fulfilling the inclusion criteria were enrolled from the indoor wards of the Orthopedic Department of Provincial Headquarter Teaching Hospital Gilgit. Informed consent was obtained from all enrolled patients. A detailed clinical history was taken, and a physical examination was performed by a trainee orthopedician. Subsequently, a blood sample was drawn for the analysis of serum albumin levels. All enrolled patients were managed conservatively with above-knee plaster casting and were discharged 48 hours after treatment with standard advice and medications prescribed on a discharge card. All treated patients were followed up four months after treatment.

Patients were evaluated for fracture union, which was defined as the presence of bridging callus over at least three cortices on standard anteroposterior and lateral radiographs, along with the absence of pain and tenderness at the fracture site. The patients were evaluated by a consultant orthopedician. Serum albumin levels were measured at the time of initial presentation and again at four months after treatment. Changes in serum albumin were compared between patients with normal union and those with impaired union of tibial diaphyseal fractures. All collected information was entered into the proforma.

Data were entered and analyzed using the IBM SPSS Statistics for Windows, Version 21.0 (Released 2012; IBM Corp., Armonk, NY, USA). Quantitative variables such as age, serum albumin levels at baseline and at four months after treatment, and the change in serum albumin levels were expressed as mean ± SD. Qualitative variables such as gender, type of tibial fracture, mode of fracture, and impaired union were expressed as frequencies and percentages. Changes in serum albumin levels were compared between patients with impaired union and those with normal union. An independent-sample t-test was applied, and a p-value ≤ 0.05 was considered statistically significant. Effect modifiers such as age, gender, type of fracture, and mode of fracture were controlled through stratification. Post-stratification independent-sample t-tests were applied, and a p-value ≤ 0.05 was considered statistically significant.

## Results

A total of 110 patients with simple diaphyseal tibial fractures were enrolled in this study. The mean age of the patients was 35.3 ± 11.8 years, and most patients were male (77.3%, n = 85). The types of diaphyseal tibial fractures observed in the study population were evenly distributed among three main categories. Transverse fractures were present in 32.7% of cases (n = 36), oblique fractures accounted for 33.6% of cases (n = 37), and spiral fractures were also observed in 33.6% of patients (n = 37). The most common etiology of injury was road traffic accidents, followed by falls from height (Table 1).

**Table 1:** Baseline characteristics of patients

Characteristic	Group A (normal union) (n = 92)	Group B (impaired union) (n = 18)	Total (n = 110)	p-Value
Age (years), mean ± SD	33.1 ± 10.5	39.8 ± 12.1	35.3 ± 11.8	0.02
Gender, n (%)				0.502
Male	70 (82.4%)	15 (17.6%)	85 (100%)	
Female	22 (88.0%)	3 (12.0%)	25 (100%)	
Mode of trauma, n (%)				0.665
Road traffic accident	66 (84.6%)	12 (15.4%)	78 (100%)	
Fall from height	26 (81.3%)	6 (18.8%)	32 (100%)	
Type of fracture, n (%)				0.569
Transverse	31 (86.1%)	5 (13.9%)	36 (100%)	
Oblique	29 (78.4%)	8 (21.6%)	37 (100%)	
Spiral	32 (86.5%)	5 (13.5%)	37 (100%)	

**Figure 1:**
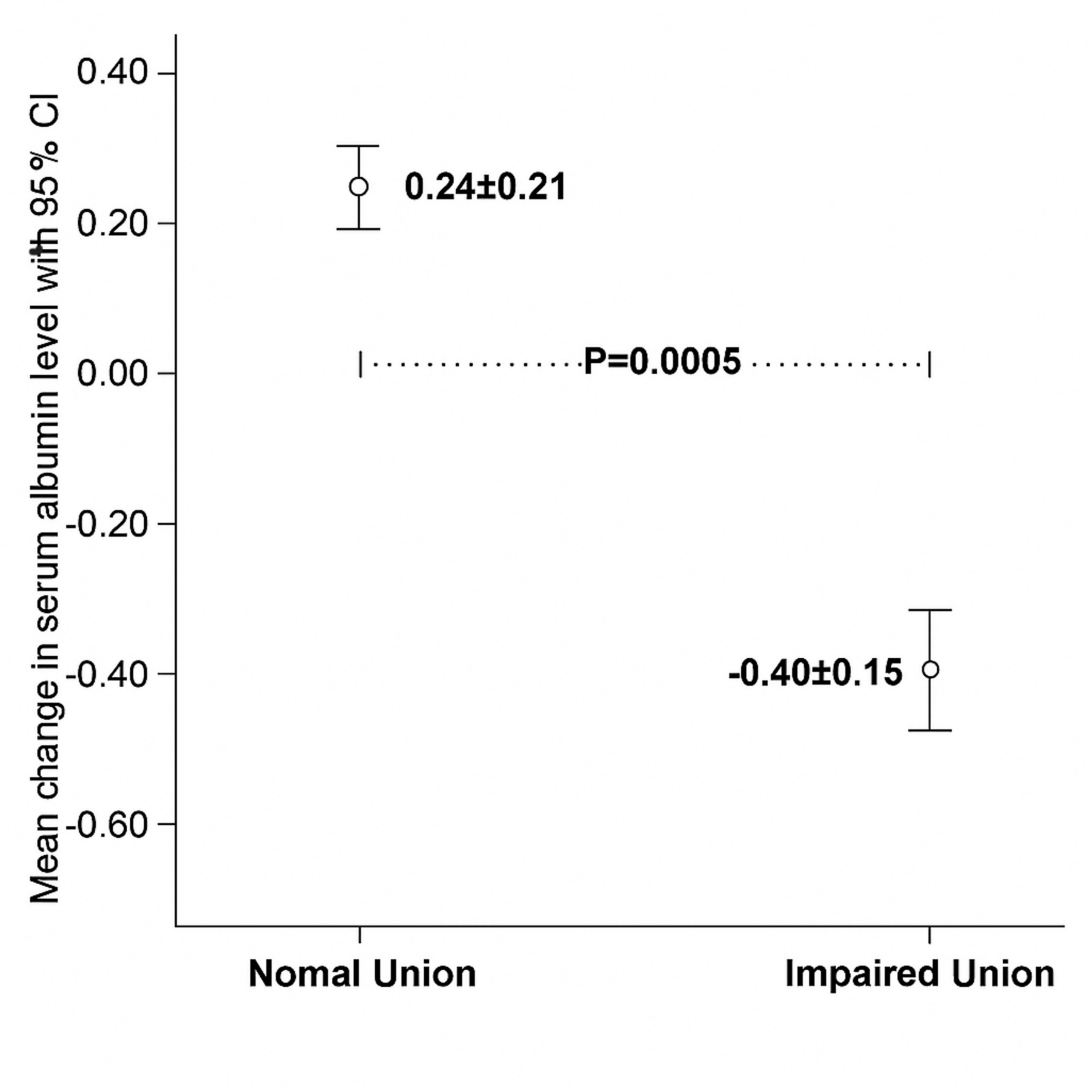
Comparison of mean change in serum albumin (g/dL) between patients with normal union and impaired union

In this study, impaired union was observed in 16.4% of patients, corresponding to 18 of the 110 individuals enrolled. A significant difference was observed in the mean change in serum albumin levels between the two groups. Patients in the Union Group demonstrated a positive mean change (0.24 ± 0.21 g/dL), whereas those in the Impaired Union Group exhibited a negative mean change (-0.40 ± 0.15 g/dL) (p < 0.001, Figure 1).

Stratified analysis showed that the rate of impaired union was significantly higher in patients aged 51-60 years. No statistically significant associations were observed between union outcomes and gender, mode of trauma, or fracture type; however, the positive mean difference in serum albumin levels in the Union Group compared with the Impaired Union Group remained consistent and statistically significant across all stratified subgroups (Table 2).

**Table 2:** Comparison of mean change in serum albumin levels stratified by age group, gender, mode of trauma, and fracture type

Stratification variable	Group A (normal union)		Group B (impaired union)		p-Value
	n	Mean change (SD)	n	Mean change (SD)	
Age groups					
<30 years	39	+0.23 (0.20)	4	-0.37 (0.14)	<0.001
31-40 years	30	+0.24 (0.22)	3	-0.40 (0.16)	<0.001
41-50 years	15	+0.29 (0.23)	2	-0.45 (0.07)	0.002
51-60 years	8	+0.18 (0.19)	9	-0.40 (0.17)	<0.001
Gender					
Male	70	+0.23 (0.21)	15	-0.40 (0.15)	<0.001
Female	22	+0.27 (0.22)	3	-0.40 (0.20)	0.001
Mode of trauma					
Road traffic accident	66	+0.26 (0.22)	12	-0.38 (0.14)	<0.001
Fall	26	+0.20 (0.19)	6	-0.43 (0.17)	<0.001
Type of fracture					
Transverse	31	+0.27 (0.23)	5	-0.38 (0.15)	<0.001
Oblique	29	+0.20 (0.18)	8	-0.44 (0.16)	<0.001
Spiral	32	+0.25 (0.22)	5	-0.36 (0.13)	<0.001

## Discussion

Tibial fracture healing is a complex process that involves multiple physiological mechanisms and is influenced by both intrinsic and extrinsic factors. Identifying patients at risk of fracture nonunion remains a key challenge for clinicians. This study provides evidence that observing changes in serum albumin levels following injury is a practical and dynamic method for anticipating the healing process in patients [12,13]. A total of 110 patients with simple diaphyseal tibial fractures, which are common in high-energy trauma, were included in the study, with a mean age of 35.3 years, and 77.3% of the population being men. Impaired union was observed in 16.4% of the study population. Another important finding was the variation in serum albumin levels between patients with successful and impaired union. The Union Group presented a positive mean change in albumin levels, whereas a significant decrease was observed in the Impaired Union Group. This distinction emphasizes that the nutritional and metabolic responses of the body following injury are closely related to bone healing.

Serum albumin is a direct indicator of nutritional status and an important factor in fracture healing. Other studies, including that by Weszl et al., have demonstrated that albumin is capable of inducing the growth of mesenchymal stem cells on bone allografts, which is a critical event in bone regeneration [14]. Increased osteoblast proliferation, which is essential for new bone formation and fracture healing, may be enhanced in patients with higher serum albumin levels. The higher levels of albumin observed in the Union Group in this study indicate a more favorable internal environment for bone regeneration [15]. This observation is consistent with previous reports showing that low albumin levels are associated with slower recovery and poorer outcomes [16,17].

These findings should be interpreted in the context of the broader scientific literature. Although some studies, such as that by Dwyer et al., did not identify a strong relationship between general nutritional status and fracture healing [18], growing evidence has emphasized the importance of specific biomarkers. For example, Ali et al. reported a relationship between serum albumin levels and fracture outcomes, regardless of whether overall nutritional status was significantly associated with the healing process [19]. The results of the present study, which demonstrate consistent and statistically significant variability in albumin changes across all subgroups of the Union and Impaired Union Groups, further support the use of serum albumin as a sensitive and reliable early predictor of healing potential.

These results have important clinical implications. The subcutaneous position of the tibia predisposes it to postoperative complications. Reliance on clinical and radiologic examinations alone may delay the detection of nonunion and prolong patient uncertainty. This study supports proactive monitoring of serum albumin changes in the post-injury period. Such monitoring may enable earlier identification of patients at risk of impaired union, particularly older patients (aged 51-60 years), who demonstrated a higher rate of impaired healing. Early identification may facilitate timely interventions, including targeted nutritional support or closer clinical observation, thereby reducing the risk and impact of nonunion.

This study has several limitations. Its observational cohort design demonstrates association but not causation between changes in serum albumin levels and fracture healing. The findings may not be generalizable due to the single-center design and limited sample size. In addition, potential confounders were not adjusted for, including detailed nutritional status (e.g., body mass index and vitamin D status), patient habits (e.g., smoking), and systemic inflammatory states that may affect serum albumin levels independently of nutrition. Furthermore, a specific clinical threshold for changes in serum albumin was not determined in this study. Patients with fractures managed surgically were also excluded.

## Conclusions

Our study demonstrates that the trajectory of serum albumin in the post-injury period is a potent predictor of healing outcomes in simple tibial fractures. A rising trend in albumin levels is a positive sign, reflecting healthy fracture union. Conversely, a declining albumin level should serve as a red flag, signaling a high risk of impaired union. Integrating this simple, cost-effective biomarker into routine clinical practice could empower surgeons to personalize patient care, intervene earlier, and ultimately improve the likelihood of a successful and timely recovery.
